# The Association between Lead Exposure and Bone Mineral Density in Childhood and Adolescence: Results from NHANES 1999–2006 and 2011–2018

**DOI:** 10.3390/nu14071523

**Published:** 2022-04-06

**Authors:** Tao Li, Yixuan Xie, Liang Wang, Guimin Huang, Yijing Cheng, Dongqing Hou, Wenqian Liu, Tong Zhang, Junting Liu

**Affiliations:** 1 Child Health Big Data Research Center, Capital Institute of Pediatrics, Beijing 100020, China; socott@126.com (T.L.); xie1xuan@hotmail.com (Y.X.); guiminhuang@163.com (G.H.); yijing_cheng@163.com (Y.C.); dqhou@sina.com (D.H.); 13811325178@163.com (W.L.); zt@chinawch.org.cn (T.Z.); 2 Department of Public Health, Robbins College of Health and Human Sciences, Baylor University, Waco, TX 76789, USA; liang_wang1@baylor.edu

**Keywords:** lead exposure, BMD, childhood, adolescence, epidemiology, NHANES

## Abstract

There are few studies on lead’s effect on bone mineral density (BMD) in childhood. In this study, we examined the association between lead exposure and BMD among 13,951 children and adolescents aged 8–19 years from NHANES 1999–2006 and 2011–2018. The whole blood lead levels (BLLs) were used as lead exposure biomarkers, and total BMD, subtotal BMD, lumbar spine BMD and limb BMD were used as outcome variables. The survey weighted multivariable generalized additive models (GAMs) with smoothing terms were used to explore the association between blood lead levels and BMDs, adjusted for age, sex, race/ethnicity, height, weight, family-income-to-poverty ratio and blood cadmium. Subgroup analyses stratified by sex and bony sites were further performed. We found an N-shaped curve association between BLLs and total BMD, subtotal BMD and limb BMD for males and females, whereas the association between BLLs and lumbar spine BMD was only significantly negative for females. The findings suggested that lead exposure had different effects on BMD of different bony sites (highly cortical or trabecular regions) in childhood and adolescence and had different effects on the same bone among different ages population and/or at different levels.

## 1. Introduction

Bone is an important component of the human body. The bones of the human body have the functions of protection, support, hematopoiesis, storage and movement. Bone mass is maintained through a balance of bone formation and resorption. Bone mineral density (BMD) is an important indicator of bone strength, which is usually used to evaluate the health status of bone. Many factors including nutrition, physical activity and life behaviors affect it [1,2]. Offspring’s BMD is even influenced by maternal vitamin D status and diet during their mother’s pregnancy anthropometry and birth weight [3]. People with osteoporosis and fracture are always with lower BMD [4]. There have been many studies about the effects of childhood exposure to lead, even at a low level, on the health of multiple human organs, such as lower cognitive function and neurobehavior, declines in IQ in childhood [5,6,7], and even the socioeconomic status in adult [8]. However, there are few studies on the effects of lead on human bone health.

There were studies conducted in the general US adult population which showed that blood lead levels (BLLs) were associated with a decreased total femur BMD, total spine BMD [9], lumbar spine BMD [10] and total BMD [11]. Blood lead concentrations tended to increase bone loss when both plasma folate and plasma vitamin B_12_ were at low levels in Taiwanese adults [12]. However, Campbell, J.R. et al.’s study on children aged 8–10 years old found that there were different trends between lead exposure and BMD, in which subjects with higher lead exposure had a significantly higher BMD of total body, lumbar spine and hip region than subjects with lower lead exposure [13]. A study conducted with adolescent mice showed that there was an unexpected increase in femur-tibial bone mass when exposed to increasing lead levels (0, 200 and 500 ppm). While the study among normal children or adolescents focusing on the effects of normal lead exposure on bone is limited.

Since the early 1960s, the National Center for Health Statistics (NCHS) of the Centers for Disease Control and Prevention (CDC) of the United States designed a program, the National Health and Nutrition Examination Survey (NHANES), to assess the health and nutritional status of adults and children in the United States. In 1999, the survey became a continuous program and began being conducted every two years. A series of surveys have been finished up to now, and BMD and BLLs are routine laboratory test content for children and adolescents in certain age groups [14]. In this study, we used data from NHANES to explore the relationship between BLLs and BMD among children and adolescents.

## 2. Methods

### 2.1. Study Design and Participants

Our initial plan was to include the last ten available surveys of NHANES (from 1999 to 2018), but the surveys of 2007–2008 and 2009–2010 were not included due to the unavailability of subtotal and lumbar BMD. Our study objects were children and adolescents, but due to BMD data were only available for participants 8 years and older in NHANES; finally, participants aged ≥ 8 and <20 years old and with valid whole blood lead concentration, total BMD, subtotal BMD, lumbar spine BMD, leg BMD and arm BMD data were involved. The participants with removable or non-removable objects, parts of the body out of the scan region, positioning problems and other problems when exanimated BMD were excluded. The data used in this study are from publicly available and deidentified data frames released by the CDC of the US. The NCHS Research Ethics Review Board reviewed and approved the study, and the protocol numbers were available at https://www.cdc.gov/nchs/nhanes/irba98.htm, accessed on 1 January 2022. Informed consent was obtained from all subjects involved in the study.

### 2.2. Measurement of Blood Lead

The lead concentration of the whole blood was chosen as the exposure biomarker and was measured using inductively coupled plasma dynamic reaction cell mass spectrometry (ICP-DRC-MS) after a simple dilution sample preparation step [15]. The lower limit of detection was 0.7 μg/L for blood lead. The measurement results below the lower limit of detection were placed by the lower limit of detection divided by the square root of 2.

### 2.3. Examination of BMD

The outcome variables we used in the study were lumbar spine BMD, limb BMD, subtotal BMD and total BMD. The dual-energy X-ray absorptiometry (DXA) examination was used to measure the BMD of participants by trained and certified radiology technologists. Further details of the DXA examination protocol are documented in the Body Composition Procedures Manual located on the NHANES website [16].

### 2.4. Statistical Analysis

Sampling weights were calculated using procedures that follow the National Center for Health Statistics Analytic Guidelines. We used geometric means and 95% confidence intervals to report the mean BLLs. This method was typically applied because of the skewed distribution of human blood lead values. After exponential transformation of the blood lead values, the distribution was normal. After the initial BLL values were ln transformed, the difference among the average BLLs was evaluated with a t-test. Pearson’s Chi-squared test was used to the proportion difference among levels of category factors. We calculated multivariable generalized additive models (GAMs) with smoothing terms. The models adjusted for age, sex, race/ethnicity, height, weight, family-income-to-poverty ratio and blood cadmium levels (BCLs) in whole participants models. Subgroup analyses stratified by sex and bony sites were further performed. When calculating the models stratified by sex, all covariates in the above models, excluding sex, were adjusted. When calculating the models stratified by bony sites, all covariates in whole-participant models were adjusted. Because there was no uniform variable on physical activity and dietary intake for children and adolescents in the survey cycles included, these factors could not be put into models as covariates. Thus, we performed a sensitivity analysis using the physical activity questionnaire data (during the past 7 days, on how many days were you physically active for a total of at least 60 min per day?) and calcium and vitamin D intakes, both of which are available for participants of certain ages. A *p*-value < 0.05 was considered statistically significant. Statistical analysis was performed using R statistical software, version 3.6.3.

## 3. Results

### 3.1. Characteristics of Participants

**Figure 1:**
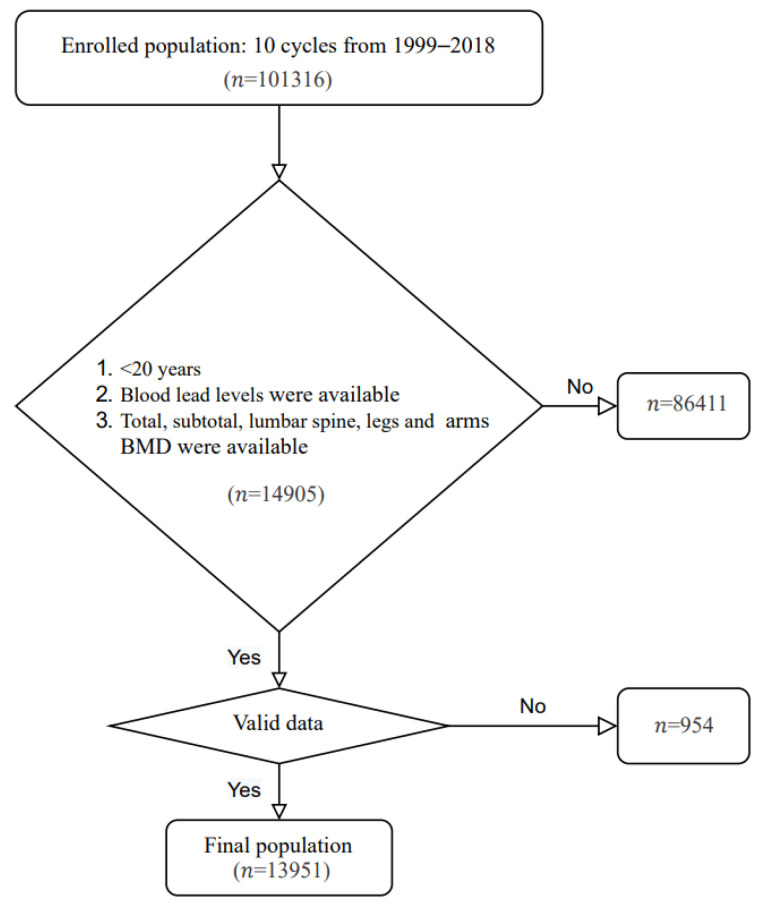
Flow chart of participants from NHANES included in the study. BMD, bone mineral density.

A total of 13,951 participants aged ≥ 8 and <20 years old were included in the study. The mean age was 13.53 ± 3.42 years (mean ± SD), and 54.8% were male. The selection flow chart is shown in Figure 1.

The characteristics of all included participants are presented in Table 1. The average BLL was 8.49 μg/L (GM 95% CI: 8.39, 8.59 μg/L), and 97.5% and 90% of the participants were less than 38.00 μg/L and 21.00 μg/L, respectively. The average values (mean ± SD) of total BMD, subtotal BMD, lumbar spine BMD and limb BMD were 0.991 ± 0.161 g/cm^2^, 0.886 ± 0.162 g/cm^2^, 0.889 ± 0.192 g/cm^2^ and 0.924 ± 0.164, respectively. The subgroup analysis stratified by sex shows that there was no significant difference in average age, race composition and the ratio of family income to poverty between males and females (*p* > 0.05 for all). However, there were statistically significant differences in height, weight, BLLs, BCLs, total BMD, subtotal BMD, lumbar spine BMD and limb BMD (*p* < 0.001 for all). Males were with higher BLLs, BCLs, total BMD, subtotal BMD and limb BMD, but lower lumbar spine BMD (*p* < 0.001 for all).

### 3.2. GAMs Analysis

#### 3.2.1. The Association between BLLs and BMD in Whole Participants Population

**Figure 2:**
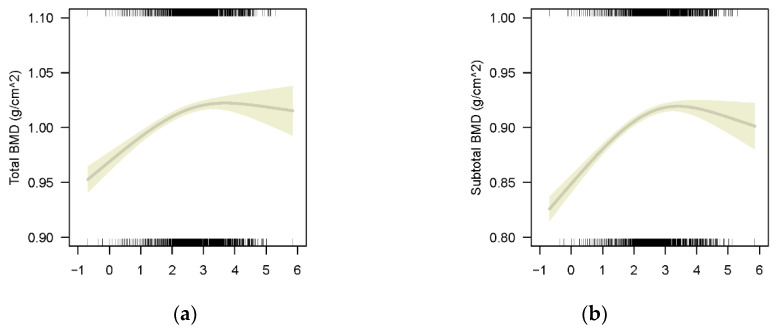
Multivariate models for ln-transformed BLLs on total BMD and subtotal BMD adjusted for age, sex, race/ethnicity, height, weight, family-income-to-poverty ratio and blood cadmium, with results weighted for sampling strategy: (**a**) Total BMD, F = 50.73, *p* < 0.001, R-sq.(adj) = 0.755; (**b**) Subtotal BMD, F = 124.2, *p* ≤ 0.001, R-sq.(adj) = 0.799.

We observed an N-shaped curve association between ln-transformed BLLs and total and subtotal BMD for the whole population (*p* < 0.001 for lumbar spine BMD and limb BMD) in weighted GAMs adjusted for age, sex, race/ethnicity, height, family income-to-poverty ratio and blood cadmium concentration. The fitted curve is shown in Figure 2. The association between ln-transformed BLLs and total BMD was positive before ln-transformed BLLs reaching 3 (the turning point of the N-shaped curve, Figure 2a), and then became negative. The corresponding BLL value of ln-transformed BLL 3 is 20 μg/L. It means that participants’ total BMD increased with BLL rising under 20 μg/L and then decreased. A similar curve is also found for subtotal BMD in Figure 2b.

#### 3.2.2. The Association between BLLs and BMD of Different Bony Sites

**Figure 3:**
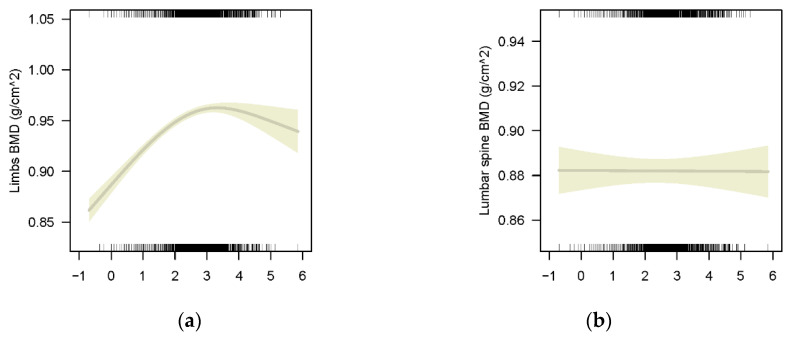
Multivariate models for ln-transformed BLLs on different bony sites’ BMD adjusted for age, sex, race/ethnicity, height, weight, family-income-to-poverty ratio and blood cadmium, with results weighted for sampling strategy: (**a**) Limb BMD, F = 151.9, *p* ≤ 0.001, R-sq.(adj) = 0.802; (**b**) Lumbar spine BMD, F = 1.554, *p* = 0.213, R-sq.(adj) = 0.681.

A similar N-shaped curve association was found between ln-transformed BLLs and limb BMD (*p* < 0.001 for all) in weighted GAMs adjusted for age, sex, race/ethnicity, height, family-income-to-poverty ratio and blood cadmium concentration. The fitted curve is shown in Figure 3a. The association between ln-transformed BLLs and limb BMD was positive also before ln-transformed BLLs reaching 3 (the turning point of the N-shaped curve) and then became negative (*p* ≤ 0.001). However, a monotonically negative association was found between lumbar spine BMD and BLLs though no statistical significance (*p* = 0.213, the curve is in Figure 3b).

#### 3.2.3. The Association between BLLs and BMD of Different Sex

**Figure 4:**
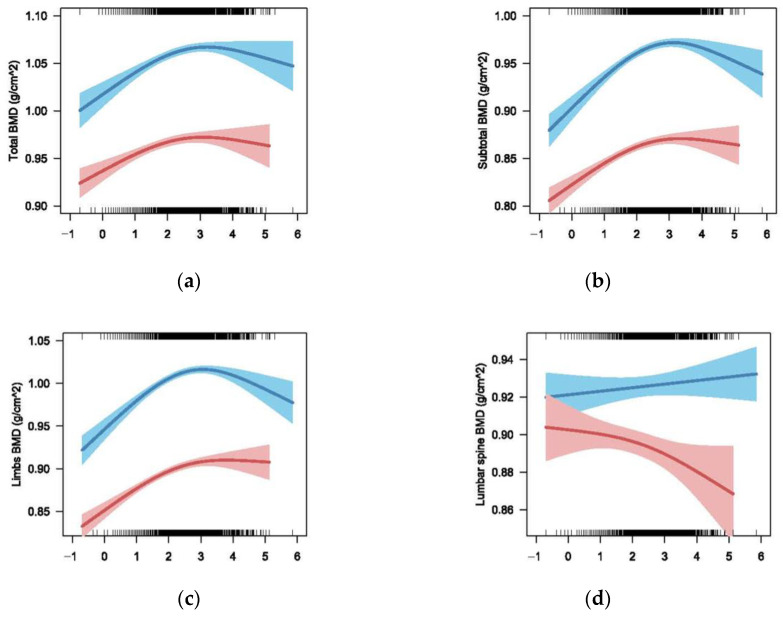
Multivariate models for ln-transformed BLLs on total BMD, subtotal BMD, limb BMD and lumbar spine BMD stratified by sex (blue line for male and red line for female) adjusted for age, race/ethnicity, height, weight, family-income-to-poverty ratio and blood cadmium, with results weighted for sampling strategy: (**a**) Total BMD: F = 29.87, *p* < 0.001, R-sq.(adj) = 0.77 for male; F = 20.38, *p* < 0.001, R-sq.(adj) = 0.731 for female; (**b**) Subtotal: F = 62.11, *p* < 0.001, R-sq.(adj) = 0.819 for male; F = 48.13, *p* < 0.001, R-sq.(adj) = 0.761 for female; (**c**) Limb BMD: F = 64.33, *p* < 0.001, R-sq.(adj) = 0.826 for male; F = 68.42, *p* < 0.001, R-sq.(adj) = 0.754 for female; (**d**) Lumbar spine: F = 0.917, *p* = 0.338, R-sq.(adj) = 0.689 for male; F = 2.965, *p* = 0.035, R-sq.(adj) = 0.663 for female.

We observed a similar N-shaped curve association between ln-transformed BLLs and total BMD, subtotal BMD and limb BMD for males and females, respectively (*p* < 0.001 for all) in weighted GAMs adjusted for age, race/ethnicity, height, family-income-to-poverty ratio and blood cadmium concentration. The fitted curves are shown in Figure 4a–c. The association between ln-transformed BLLs and BMD was positive before ln-transformed BLLs, reaching approximately 3 (the turning point of the N-shaped curve), and then became negative. The turning point of females seemed earlier than that of males for all three types of BMD, whereas, in the models with lumbar spine BMD, a statistically significant decreasing trend was found for females (*p* = 0.035) but not for males (*p* = 0.338).

### 3.3. Sensitivity Analysis

Participants with both variables of physical activity and calcium and vitamin D intakes were selected from our study population. After participants aged 12 years and older were excluded due to the small sample size, 2116 participants aged 8–11 years old were included for the sensitivity analysis. We conducted two models for each bony site BMD (total, subtotal, limbs and lumbar spine) among subgroup participants. In the first model, age, sex, race/ethnicity, height, weight, family-income-to-poverty ratio and blood cadmium were adjusted. In the second model, physical activity and dietary calcium and vitamin D intake plus variables in model 1 were considered. However, the curve shapes were consistent, and no significant change was observed between the two models (see Supplement Figure S1).

## 4. Discussion

In this study, we examined the association between BLLs and BMDs among a representative sample of the US child and adolescent population from NHANES 1999–2006 and 2011–2018. We found BLLs had an N-shaped curve association with total BMD, subtotal BMD and limb BMD for males and females, whereas the association between BLLs and lumbar spine BMD was only significantly negative for females.

First of all, the lumbar spine BMD decreased with BLLs rising, but limb BMD increased before BLLs reached the turning point of 20 μg/L. Thus, we speculated that lead might have different effects on BMD of different bony sites of children and adolescents at certain levels.

One study conducted in chick growth plate and sternal chondrocyte models found that lead impacts the modulation progress of chondrocyte maturation by inhibiting the parathyroid hormone-related peptide (PTHrP) and transforming growth factors and results in accelerating bone maturity [17]. (The N-terminus and the secondary structure of multiple isoforms of PTHrP resemble the parathyroid hormone (PTH), allowing PTHrP to bind to the same receptor as PTH and to have some similar effects, including increasing bone resorption and calcium reabsorption in the distal renal tubules and inhibiting phosphate transport in the proximal tubules.) A study on postmenopausal women with osteoporosis showed that once-daily administration of recombinant human parathyroid hormone (1–34) (Teriparatide) induced beneficial changes in the structural architecture of distal radial diaphysis, with an increase in total bone mineral content, but without significant effects on cortical bone content, total BMD or cortical BMD [18]. The cancellous bone has a greater surface area compared with cortical bone and consequently is ideal for metabolic activity, and lead is selectively taken up at trabecular bone compared with cortical bone [19]. Therefore, we speculated that if PTH/PTHrP was inhibited by lead toxicity in humans, it might mainly reduce BMD of highly trabecular sites, such as the lumbar spine, and had little effect on largely cortical regions, such as limbs.

In another study on adolescent mice, higher lead exposure increases trabecular bone mass, cortical thickness and BMD in femurs (largely cortical regions) but results in no change in BMD in the vertebras (highly trabecular sites) by depressing the ability of osteoclasts to resorb bone mass [20].

Another finding in our study was the N-shaped curve association between BLLs and total/subtotal BMD, which was different in adults [11], but the reason is not clear. It might be due to changes in hormonal levels at different ages. Further metabolism studies related to bony sites differences in bone remodeling, osteoclastic bone resorption and bone formation capability under different lead exposure levels and among different age populations are warranted.

Lumbar spine BMD of females seems to be more sensitive to the effect of blood lead compared to other bony sites’ BMD. In our study, lumbar spine BMD decreased even at low lead exposure levels in females, but some other bony sites’ BMD started to decrease until the BLLs reached a certain level (20 µg/L). The negative association between BLLs and lumbar spine BMD among females but not among males in our study was consistent with results in adult studies of NHANES, in which BLLs were associated with decreased total spine BMD among non-menopausal women [9] or lumbar spine BMD among women [10,21]. A similar association was also found among Korean women [22]. However, there was also an insignificant association found in another study conducted in 50–70 years old women [23]. The lead affected follicles in mice even at low levels [24], and the follicles are the main source of endogenous estrogen. Women experience a rapid loss of bone during the first 5–10 years after menopause due to the decrease in estrogen [25]. Thus, we speculated that lead exposure induces a decrease in BMD by suddenly lowering the estrogen level. Wang et al. thought that no statistically significant association in menopausal women was due to the much weaker effect of lead on BMD compared to estrogen [9]. The reason why the association among men was not significant might be due to men’s higher peak bone mass than women in early adulthood.

Another reason for the decrease in limb BMD, total BMD and subtotal BMD when the BLLs were higher than 20 µg/L was the unstable results caused by the small sample size of the participants. In our study, only 10% of the participants were with BLLs higher than 20 µg/L, and the confidence interval was wide (Figure 3 and Figure 4).

While in Campbell, J.R. et al.’s study, the subjects with higher lead exposure (mean, 236 µg/L) had a significantly higher BMD of the lumbar spine than the subjects with lower lead exposure (mean, 65 µg/L) [13], which is contrary to the conclusion of the previous studies from adults and our study. However, the study sample was small, and the exposure levels were much higher. In the participants of our study, 97.5% BLLs were less than 38 µg/L, which was much lower than that in Campbell, J.R. et al.’s study. The discrepancy in blood lead’s effect on lumbar spine BMD prompts us to speculate that blood lead affects bone mass or BMD differently for children and adolescents at different levels.

The main strength of the study was that it was a representative epidemiology study among a child and adolescent population with the biggest sample size up to now and had enough tolerability to conduct subgroup analysis and to adjust for potential confounders.

There were several limitations in our study. First, the BLL in the blood was the only lead exposure biomarker, and bone lead, which is another lead exposure marker, was not used due to a lack of data in NHANES. Second, children younger than 8 years old were not enrolled in our study because there were no BMD data for those children; thus, we cannot explore the effects of lead on BMD in early childhood. Third, many factors including nutrition, physical activity and life behaviors affect human BMD. Because there was no uniform data on physical activity and nutrition variables for children and adolescents in the included survey cycles, they could not be put into the final models as covariates. However, a sensitivity analysis among subgroups did not find any differences between models that included physical activity and dietary factors and those that did not. More studies with detailed data on physical activity, dietary intake, other lifestyle factors and birth-related factors are needed.

## 5. Conclusions

In summary, lead toxicity had inconsistent effects on BMDs of different human bony sites and inconsistent effects on BMDs of the same bony site but in different age or sex populations or at different exposure levels. Further prospective and experimental studies are worth conducting to verify our findings and clarify the underlying biological mechanisms.

## Figures and Tables

**Table 1:** Characteristics of included participants *.

Characteristics	Total (*n* = 13,951)	Male (*n* = 7647)	Female (*n*= 6304)	*p*
Age (years, mean ± SD)	13.53 ± 3.42	13.56 ± 3.40	13.49 ± 3.45	0.229
Race (%)				
Mexican American	29.0	29.0	29.0	0.214
Other Hispanic	6.2	6.0	6.4	
Non-Hispanic White	26.4	26.2	26.7	
Non-Hispanic Black	29.5	30.2	28.6	
Other Race	8.9	8.6	9.3	
Height (cm, mean ± SD)	157.6 ± 15.1	160.5 ± 16.7	154.0 ± 12.1	<0.001
Weight (kg, mean ± SD)	57.1 ± 20.5	58.8 ± 21.7	55.1 ± 18.7	<0.001
Blood Lab test (μg/L, GM 95% CI)				
Lead	8.49 (8.39, 8.59)	9.97 (9.81, 10.13)	6.99 (6.87, 7.10)	<0.001
Cadmium	0.18 (0.18, 0.19)	0.19 (0.19, 0.19)	0.18 (0.18, 0.18)	<0.001
BMD (g/cm^2^, mean ± SD)				
Total	0.991 ± 0.161	1.002 ± 0.170	0.977 ± 0.148	<0.001
Subtotal	0.886 ± 0.162	0.904 ± 0.178	0.863 ± 0.137	<0.001
Lumbar Spine	0.889 ± 0.192	0.865 ± 0.195	0.917 ± 0.183	<0.001
Limbs ^#^	0.924 ± 0.164	0.948 ± 0.180	0.894± 0.136	<0.001
Ratio of family income to poverty (mean ± SD)	2.0 ± 1.5	2.0 ± 1.5	2.0 ± 1.5	0.484

* Data from NHANES 1999–2018 (2007–2008 and 2009–2010 were not included due to the unavailability of subtotal and lumbar BMD, so, finally, 8 cycles were included.). ^#^ The limbs include left and right arms and legs. GM, geometric mean; SD, standard deviation.

## Data Availability

All relevant data are available at https://wwwn.cdc.gov/nchs/nhanes, accessed on 1 January 2022.

## Supplementary Materials

The following supporting information can be downloaded at: https://www.mdpi.com/article/10.3390/nu14071523/s1, Figure S1: Multivariate models for ln transformed BLLs on total BMD, subtotal BMD, limbs BMD and lumbar spine BMD. In model 1 (a): Age, sex, race/ethnicity, height, weight, family income to poverty ratio and blood cadmium were adjusted. In model 2 (b): Variables in model 1 plus physical activity and dietary calcium and vitamin D intake were adjusted; Table S1: Sensitive analysis for physical activity and dietary on BMDs (n = 2116, 2011–2018, 8–11 years old).

## References

[1] Sioen I., Michels N., Polfliet C., De Smet S., D’Haese S., Roggen I., Deschepper J., Goemaere S., Valtuena J., De Henauw S. (2015). The influence of dairy consumption, sedentary behaviour and physical activity on bone mass in Flemish children: A cross-sectional study. BMC Public Health.

[2] Lappe J.M., Watson P., Gilsanz V., Hangartner T., Kalkwarf H.J., Oberfield S., Shepherd J., Winer K.K., Zemel B. (2015). The longitudinal effects of physical activity and dietary calcium on bone mass accrual across stages of pubertal development. J. Bone Miner. Res..

[3] Masztalerz-Kozubek D., Zielinska-Pukos M.A., Hamulka J. (2021). Maternal Diet, Nutritional Status, and Birth-Related Factors Influencing Offspring’s Bone Mineral Density: A Narrative Review of Observational, Cohort, and Randomized Controlled Trials. Nutrients.

[4] Deng H.W., Xu F.H., Davies K.M., Heaney R., Recker R.R. (2002). Differences in bone mineral density, bone mineral content, and bone areal size in fracturing and non-fracturing women, and their interrelationships at the spine and hip. J. Bone Miner. Metab..

[5] Boyle J., Yeter D., Aschner M., Wheeler D.C. (2021). Estimated IQ points and lifetime earnings lost to early childhood blood lead levels in the United States. Sci. Total Environ..

[6] Tatsuta N., Nakai K., Kasanuma Y., Iwai-Shimada M., Sakamoto M., Murata K., Satoh H. (2020). Prenatal and postnatal lead exposures and intellectual development among 12-year-old Japanese children. Environ. Res..

[7] Lanphear B.P., Hornung R., Khoury J., Yolton K., Baghurst P., Bellinger D.C., Canfield R.L., Dietrich K.N., Bornschein R., Greene T. (2005). Low-level environmental lead exposure and children’s intellectual function: An international pooled analysis. Environ. Health Perspect..

[8] Reuben A., Caspi A., Belsky D.W., Broadbent J., Harrington H., Sugden K., Houts R.M., Ramrakha S., Poulton R., Moffitt T.E. (2017). Association of Childhood Blood Lead Levels With Cognitive Function and Socioeconomic Status at Age 38 Years and With IQ Change and Socioeconomic Mobility Between Childhood and Adulthood. JAMA.

[9] Wang W.J., Wu C.C., Jung W.T., Lin C.Y. (2019). The associations among lead exposure, bone mineral density, and FRAX score: NHANES, 2013 to 2014. Bone.

[10] Lu J., Lan J., Li X., Zhu Z. (2021). Blood lead and cadmium levels are negatively associated with bone mineral density in young female adults. Arch Public Health.

[11] Wei M.H., Cui Y., Zhou H.L., Song W.J., Di D.S., Zhang R.Y., Huang Q., Liu J.A., Wang Q. (2021). Associations of multiple metals with bone mineral density: A population-based study in US adults. Chemosphere.

[12] Hsieh R.L., Huang Y.L., Chen W.J., Chen H.H., Shiue H.S., Lin Y.C., Hsueh Y.M. (2022). Associations between Plasma Folate and Vitamin B12, Blood Lead, and Bone Mineral Density among Adults and Elderly Who Received a Health Examination. Nutrients.

[13] Campbell J.R., Rosier R.N., Novotny L., Puzas J.E. (2004). The association between environmental lead exposure and bone density in children. Environ. Health Perspect..

[14] CDC (2017). National Health and Nutrition Examination Survey. https://www.cdc.gov/nchs/nhanes/about_nhanes.htm.

[15] CDC (2018). Cadmium, Lead, Manganese, Mercury, and Selenium Lab Procedure Manual. https://wwwn.cdc.gov/nchs/data/nhanes/2017-2018/labmethods/PBCD-J-PBY-J-R-MET-508.pdf.

[16] CDC (2013). Body Composition Procedures Manual. https://www.cdc.gov/nchs/data/nhanes/nhanes_13_14/2013_Body_Composition_DXA.pdf.

[17] Zuscik M.J., Pateder D.B., Edward Puzas J., Schwarz E.M., Rosier R.N., O’Keefe R.J. (2002). Lead alters parathyroid hormone-related peptide and transforming growth factor-β1 effects and AP-1 and NF-κKB signaling in chondrocytes. J. Orthop. Res..

[18] Zanchetta J.R., Bogado C.E., Ferretti J.L., Wang O., Wilson M.G., Sato M., Gaich G.A., Dalsky G.P., Myers S.L. (2003). Effects of teriparatide [recombinant human parathyroid hormone (1-34)] on cortical bone in postmenopausal women with osteoporosis. J. Bone Miner. Res..

[19] Inskip M.J., Franklin C.A., Subramanian K.S., Blenkinsop J., Wandelmaier F. (1992). Sampling of cortical and trabecular bone for lead analysis: Method development in a study of lead mobilization during pregnancy. Neurotoxicology.

[20] Beier E.E., Holz J.D., Sheu T.J., Puzas J.E. (2016). Elevated Lifetime Lead Exposure Impedes Osteoclast Activity and Produces an Increase in Bone Mass in Adolescent Mice. Toxicol. Sci. Off. J. Soc. Toxicol..

[21] Potula V., Kleinbaum D., Kaye W. (2006). Lead exposure and spine bone mineral density. J. Occup. Environ. Med..

[22] Lim H.S., Lee H.H., Kim T.H., Lee B.R. (2016). Relationship between Heavy Metal Exposure and Bone Mineral Density in Korean Adult. J. Bone Metab..

[23] Theppeang K., Glass T.A., Bandeen-Roche K., Todd A.C., Rohde C.A., Links J.M., Schwartz B.S. (2008). Associations of bone mineral density and lead levels in blood, tibia, and patella in urban-dwelling women. Environ. Health Perspect..

[24] Junaid M., Chowdhuri D.K., Narayan R., Shanker R., Saxena D.K. (1997). Lead-induced changes in ovarian follicular development and maturation in mice. J. Toxicol. Environ. Health.

[25] Manolagas S.C. (2000). Birth and death of bone cells: Basic regulatory mechanisms and implications for the pathogenesis and treatment of osteoporosis. Endocr. Rev..

