# Supplementary material for: The Association between Lead Exposure and Bone Mineral Density in Childhood and Adolescence: Results from NHANES 1999–2006 and 2011–2018
Source: Nutrients. 2022 Apr 6;14(7):1523. doi: 10.3390/nu14071523 (PMC9003082; doi:10.3390/nu14071523)
Supplement: Supplementary file 1 [file nutrients-14-01523-s001.zip › nutrients-1654257-SI.pdf]

Supplementary Materials

Table S1 Sensitive analysis for physical activity and dietary on BMDs (n=2116, 2011-2018, 8-11 years old)

BMD	Model 1			Model 2		
	F	P	R-sq.(adj)	F	P	R-sq.(adj)
Total	4.829	<0.001	0.516	4.068	0.0164	0.521
Subtotal	8.397	<0.001	0.676	6.18	<0.001	0.681
Limbs	11.51	<0.001	0.693	8.528	<0.001	0.698
Lumbar	0.609	0.382	0.435	1.612	0.137	0.439

In model 1, age, sex, race/ethnicity, height, weight, family income to poverty ratio and blood cadmium were adjusted.

In model 2. variables in model 1 plus physical activity and dietary calcium and vitamin D intake were adjusted.

Figure S1 Multivariate models for ln transformed BLLs on total BMD, subtotal BMD, limbs BMD and lumbar spine BMD. In model 1 (a): Age, sex, race/ethnicity, height, weight, family income to poverty ratio and blood cadmium were adjusted. In model 2 (b): Variables in model 1 plus physical activity and dietary calcium and vitamin D intake were adjusted.

(a) Model 1 (b) Model 2

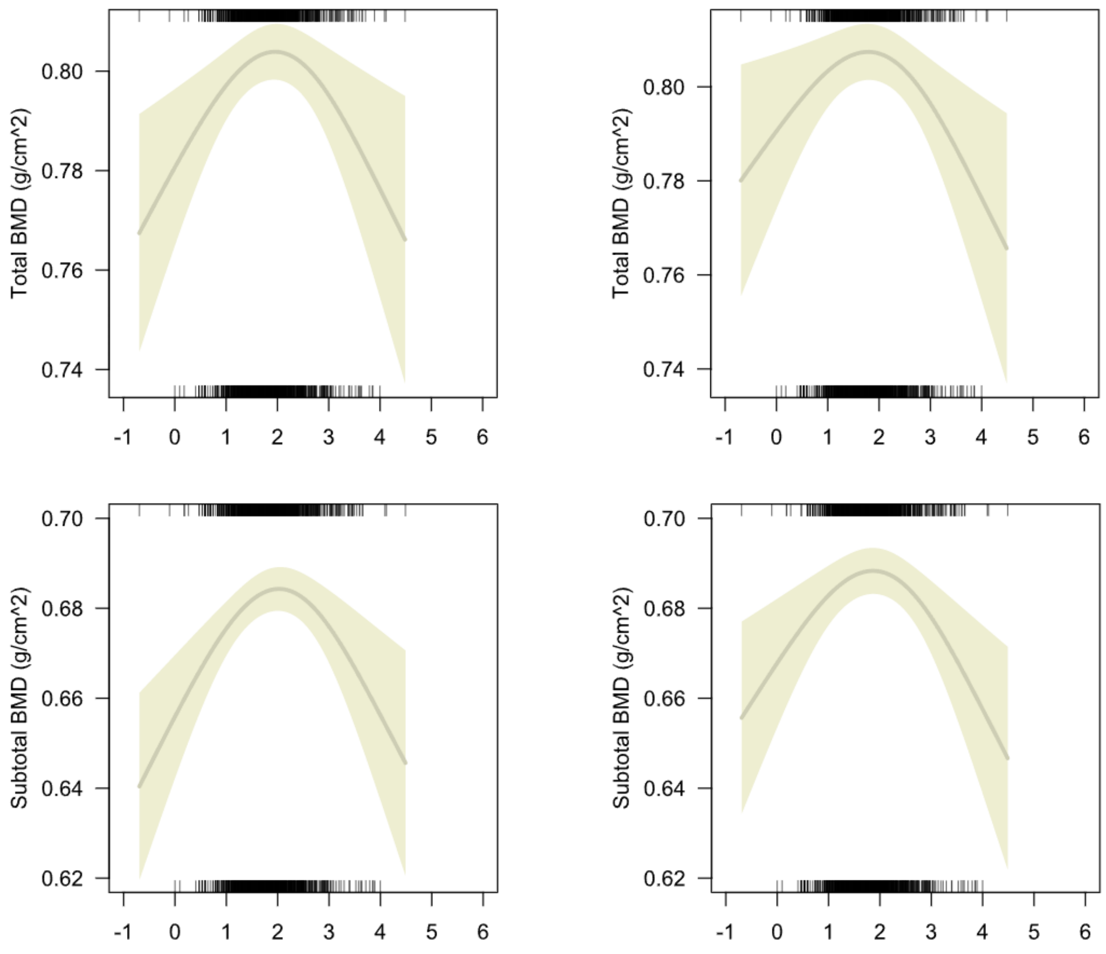

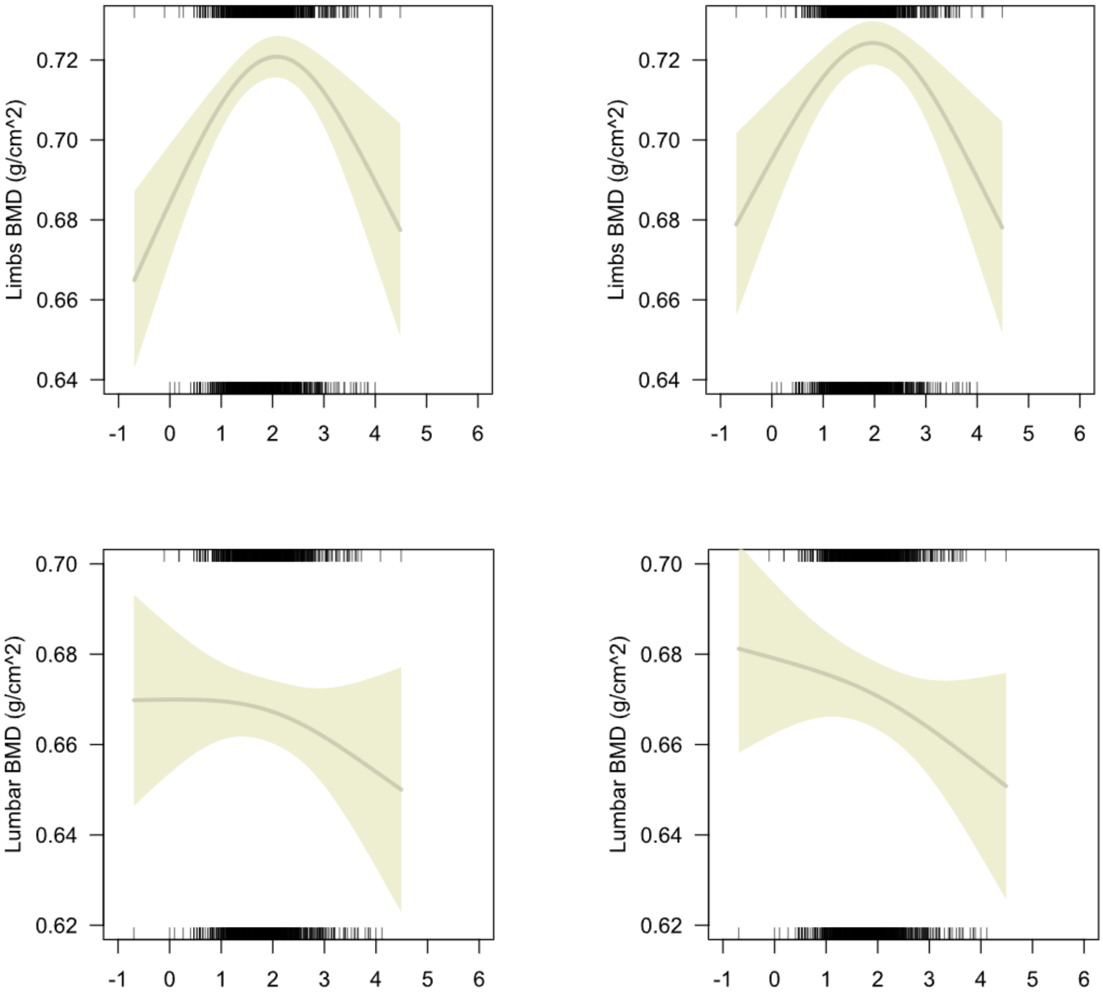
Limbs BMD (g/cm^2)
0.64
0.66
0.68
0.70
0.72
-1
0
1
2
3
4
5
6
Lumbar BMD (g/cm^2)
0.62
0.64
0.66
0.68
0.70
